# *Cuscutae semen* alleviates CUS-induced depression-like behaviors in mice *via* the gut microbiota-neuroinflammation axis

**DOI:** 10.3389/fphar.2023.1107781

**Published:** 2023-02-24

**Authors:** Lanwei Hou, Liu Yang, Caiting Zhu, Jingyu Miao, Wenjuan Zhou, Yuchun Tang, Haiwei Meng, Shuwei Liu

**Affiliations:** Department of Anatomy and Neurobiology, Research Center for Sectional and Imaging Anatomy, Shandong Provincial Key Laboratory of Mental Disorder, Shandong Key Laboratory of Digital Human and Clinical Anatomy, School of Basic Medical Sciences, Cheeloo College of Medicine, Shandong University, Institute of Brain and Brain-Inspired Science, Shandong University, Jinan, Shandong, China

**Keywords:** cuscutae semen, depression, neuroinflammation, gut microbiota, NLPR3

## Abstract

**Introduction:** Major depressive disorder is a mental disease with complex pathogenesis and treatment mechanisms involving changes in both the gut microbiota and neuroinflammation. Cuscutae Semen (CS), also known as Chinese Dodder seed, is a medicinal herb that exerts several pharmacological effects. These include neuroprotection, anti-neuroinflammation, the repair of synaptic damage, and the alleviation of oxidative stress. However, whether CuscutaeSemen exerts an antidepressant effect remains unknown.

**Methods:** In this study, we evaluated the effect of CS on chronic unpredictable stress (CUS)-induced depression-like behaviors in mice by observing changes in several inflammatory markers, including proinflammatory cytokines, inflammatory proteins, and gliocyte activation. Meanwhile, changes in the gut microbiota were analyzed based on 16 S rRNA sequencing results. Moreover, the effect of CS on the synaptic ultrastructure was detected by transmission electron microscopy.

**Results:** We found that the CS extract was rich in chlorogenic acid and hypericin. And CS relieved depression-like behaviors in mice exposed to CUS. Increased levels of cytokines (IL-1β and TNF-α) and inflammatory proteins (NLRP3, NF-κB, and COX-2) induced by CUS were reversed after CS administration. The number of astrocytes and microglia increased after CUS exposure, whereas they decreased after CS treatment. Meanwhile, CS could change the structure of the gut microbiota and increase the relative abundance of Lactobacillus. Moreover, there was a significant relationship between several Lactobacilli and indicators of depression-like behaviors and inflammation. There was a decrease in postsynaptic density after exposure to CUS, and this change was alleviated after CS treatme.

**Conclusion:** This study found that CS treatment ameliorated CUS-induced depression-like behaviors and synaptic structural defects in mice *via* the gut microbiota-neuroinflammation axis. And chlorogenic acid and hypericin may be the main active substances for CS to exert antidepressant effects.

## 1 Introduction

Major depressive disorder (MDD) is a common and multifactorial psychiatric disorder characterized by low mood and anhedonia. More than 350 million people suffer from MDD worldwide (Smith, 2014), and it has become the second leading cause of disability, as measured by years lived with disability (Whiteford et al., 2015). Currently, the limited therapeutic options available for treating MDD, such as serotonin in uptake inhibitors, are predicated on the monoaminergic neurotransmission hypothesis of depression. Unfortunately, such therapies have several shortcomings, including the delayed onset of clinical improvement (at least 2–4 weeks to take effect), inevitable side effects, and limited clinical response rates (only approximately 50% of patients show complete remission) (Deussing and Arzt, 2018). Thus, exploring new mechanisms and effective therapeutic options to treat MDD can assist in overcoming the persistent lack of progress in pharmacotherapy.

The gut microbiota-brain axis, which is a newly acknowledged system involved in the pathophysiology of MDD (Evrensel and Tarhan, 2021), has some association with neuroinflammation (Agirman et al., 2021), stress reactivity (Foster and McVey Neufeld, 2013), and host behavior (Socala et al., 2021) based on the bidirectional communication that occurs between the gut and brain. One study found that patients suffering from MDD showed changes in fecal α-diversity, including increased abundances/relative abundances of Enterobacteriaceae and *Aliella* alongside a decreased abundance/relative abundance of *Faecalibacterium* (Jiang et al., 2015). In a randomized clinical trial, Kazemi A found that probiotic supplementation (with *Lactobacillus helveticus* and *Bifidobacterium longum*) significantly reduced the Beck Depression Inventory scores and the kynurenine/tryptophan ratio of MDD patients (Kazemi et al., 2019). Animal experiments have shown that the relative abundances of *Prevotella*, *Blautia*, and *Phascolarctobacterium* changed in mice exposed to chronic unpredictable stress (CUS) (Jianguo et al., 2019). Further, the application of *Lactobacillus casei* was found to improve depression-like behaviors by modulating the BDNF-TrkB signaling pathway and intestinal microbiota (Gu et al., 2020). Therefore, exploring the role of the gut microbiota in depression is expected to provide new insights into its pharmacotherapy.

Neuroinflammatory mechanisms mediate stress to increase both emotional reactivity and depression susceptibility, and thus are considered to be important in the pathophysiology of MDD and antidepressant response (Kohler et al., 2016; Troubat et al., 2021). Clinical studies have shown that patients suffering from MDD exhibit higher levels of several proinflammatory cytokines, including interleukin (IL)-1, IL-6, and tumor necrosis factor-alpha (TNF-α) (Slavich and Irwin, 2014). These cytokines activate the hypothalamic pituitary adrenal (HPA) axis, which in turn, can induce symptoms of depression (Rosenblat et al., 2014). It has also been reported the Nod-like receptor pyrin containing three inflammasome (NLRP3), which is one of the key components involved in activating inflammatory cytokines, is increased in the peripheral blood mononuclear cells of patients suffering from MDD, while its level is decreased by the tricyclic antidepressant amitriptyline (Alcocer-Gomez et al., 2014). It is noteworthy that regulating neuroinflammatory disturbance is an important means by which the gut microbiota can induce remission from depression. Aggregated evidence from human and animal studies suggests that probiotics significantly reduce the circulating levels of inflammatory markers, and this is thought to be a key mechanism underlying their antidepressant effect (Pinto-Sanchez et al., 2017; Guo et al., 2019; Reininghaus et al., 2020). Further, many antidepressants, such as ketamine and melatonin, also exert their antidepressant effects by regulating the gut microbiota-neuroinflammation axis (Lv et al., 2020; Wang et al., 2021). Thus, modulating the gut microbiota-neuroinflammation axis might comprise a novel strategy for the treatment of MDD.

*Cuscutae Semen* (CS), which refers to seeds produced by the *Cuscuta chinensis* Lam. plant, contains high contents of flavonoids, polysaccharides, alkaloids, and resin glycosides. Several studies have shown that CS has various biological activities pertaining to neuroprotection (Zhen et al., 2006), anti-neuroinflammation (Kang et al., 2014), the rescue of synaptic damage (Ju et al., 2019), and the alleviation of oxidative stress (Lin et al., 2018). Seok Y et al. reported that CS extract suppresses the inflammatory response by inhibiting the expression of c*yclooxygenase*-*2* (COX-2), downregulating the transcription levels of TNF-α, IL-1β, and IL-6, and contributing to the nuclear translocation of nuclear factor kappa B (NF-κB) P65 in activated microglia (Kang et al., 2014). Meanwhile, CS has been found to inhibit the phosphorylation of both glycogen synthase kinase-3β and tau proteins, which then contribute to synaptic dysfunction (Ju et al., 2019). However, whether CS acts as an antidepressant through the gut microbiota-neuroinflammation axis has remained unknown.

From the available evidence, we hypothesized that CS acts as an antidepressant in mice exposed to CUS, and that the gut microbiota-neuroinflammation axis might mediate this effect. Hence, we induced depression-like behaviors in mice by subjecting them to CUS. Then, the ability of CS to alleviate depressive behaviors was determined by conducting behavioral tests, while the characteristics of the gut microbiota and neuroinflammation of the mice were measured by 16rs sequencing and biochemical tests, respectively.

## 2 Materials and methods

### 2.1 Animals

Fifty-eight male C57BL/6N mice (6-week-old, weight 20 ± 2 g) were utilized in this study and purchased from the animal center at Shandong University (Jinan, Shandong). The animals were housed at five mice per cage (12 h light/dark cycle) under pathogen-free conditions at constant temperature (23°C ± 1°C) and relative humidity (45% ± 10%). Food and water were available *ad libitum*. After being habituated to these conditions for 7 days, all animals underwent the experimental tests. All experiments were conducted according to the National Institutes of Health Guidelines (Use of Laboratory Animals) and were approved by the Shandong University Animal Care and Use Committees.

### 2.2 Preparation of the CS extract and quantitative analysis

The CS extract used in the present study was purchased from Chengdu DeSiTe Biological Technology Co., Ltd. (Chengdu, China). The extract was prepared by the following procedure. Briefly, CS was crushed and passed through a No. 4 pharmacopeia sieve. Then, the CS was extracted three times using 80% aqueous ethanol (CS: ethanol = 1:6) at 60°C. The extract was then combined and filtered to obtain 160 L of CS filter liquor. The filtered liquor was subsequently concentrated under reduced pressure at 60°C until no ethanol was detected to obtain the resulting concentrated solution, which was continuously concentrated and dried under reduced pressure at 60°C to obtain the final CS extract. CS and seven standards (quercetin, isoquercetin, astragalin, hypericin, kaempferol, chlorogenic acid, and isorhamnetin) were analyzed by using a HPLC system (Waters 2,695, Waters Corporation, Massachusetts, United States) and Phytochemical profiles of CS extracts were determined and compared to confirm their identities (Supplementary Figure S1; Supplementary Table S1).

### 2.3 CS toxicity test

Ten male C57BL/6N mice (6-week-old, weight 20 ± 2 g) in the study were utilized to analyze the toxicity of CS. Mice were treated by the CS extract (1.5 g/kg, 10 times the maximum dose used in this study, i. g.) for 10 days. The mice were observed for death or abnormal behavior, including hyperactivity, tremors, ataxia, convulsions, salivation, diarrhea, drowsiness, sleep, and coma.

### 2.4 Experimental design

**FIGURE 1:**
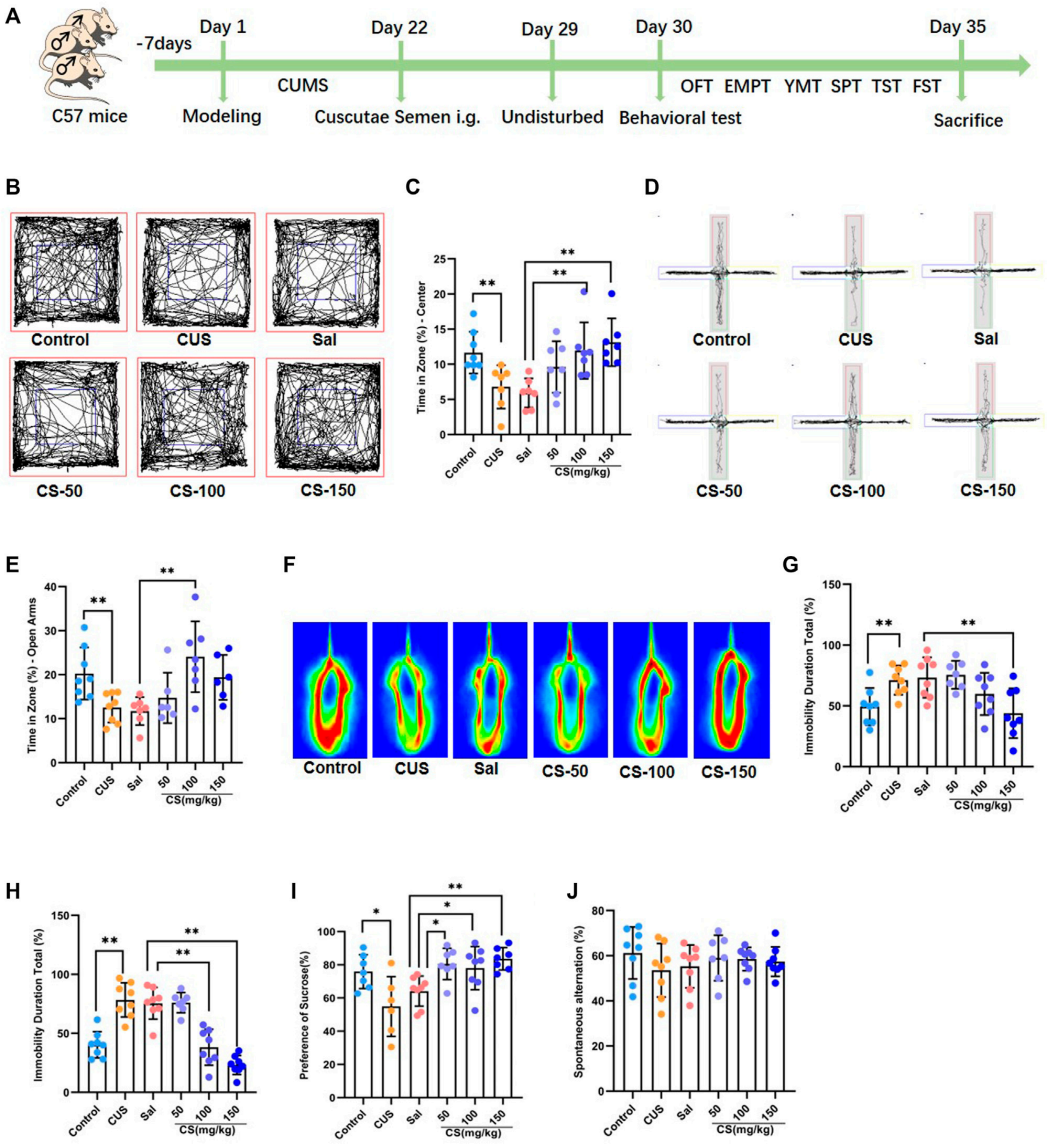
CS relieved depression-like behaviors in mice exposed to CUS. **(A)** Experimental procedure. **(B, C)** The motion trail and time spent in the center zone in the OFT. **(D, E)** The motion trail and time spent in the open arms in the EPMT. **(F, G)** The percentage of immobility time in the TST. **(H)** The percentage of immobility time in the FST. **(I)** The percentage of sucrose in the SPT. **(J)** Spontaneous alternation in the FST. Data are expressed as the mean ± standard error. *p < 0.05, **p < 0.01.

Mice were randomly divided into the following six groups: the control group (*n* = 8), in which the mice received no treatment and remained in their home cages for 21 days; the CUS group (*n* = 8), in which the mice were exposed to CUS for 21 consecutive days; the Sal group (*n* = 8), in which, after being exposed to CUS for 21 days, the mice were treated by Sal (100 mg/kg, i. g.) for 7 days; the CS-50 group (*n* = 8), in which, after being exposed to CUS for 21 days, the mice were treated with CS extract (50 mg/kg, i. g.) for 7 days; the CS-100 group (*n* = 8), in which, after being exposed to CUS for 21 days, the mice were treated with CS extract (100 mg/kg, i. g.) for 7 days; the CS-150 group (*n* = 8), in which, after exposure to CUS for 21 days, the mice were treated with the CS extract (150 mg/kg, i. g.) for 7 days. The experimental procedure is shown in Figure 1A, and the CUS procedure is shown in Supplementary Table S2.

### 2.5 Behavioral tests

After drug treatment, the mice were sequentially subjected to a series of behavioral tests, including an open-field experiment, including an open field test (OFT), elevated plus maze test (EPMT), Y-maze test (YMT), sucrose preference test (SPT), tail suspension test (TST), and Forced swimming test (FST). All the behavioral tests were carried out during the light phase (9:00–21:00) on days 30–35. And the interval between behavioral tests was 24 h.

#### 2.5.1 Open-field test

The open field test (OFT) was used to assess anxiety-like behaviors. The open field area in this experiment was an enclosed area surrounded by black plexiglass (60 × 60 × 30 cm). Each mouse was gently placed in the same corner of the arena facing the same direction and allowed to freely explore the arena for 10 min. The time spent in the center square and track movements of mice were recorded by a video camera system (SMART 3.0; Panlab S. L., Barcelona, Spain). The criterion for entering the center square was defined as 50% of the body being positioned within this square.

#### 2.5.2 Elevated plus maze test

The elevated plus maze test (EPMT) was used to assess anxiety-like behaviors. The apparatus used in this experiment included two closed arms (50 × 10 cm, with 40 cm walls), two open arms (50 × 10 cm, without walls), and a center platform (10 × 10 cm). Each mouse was gently placed on the central platform facing the closed arms and was allowed to freely explore the arena for 6 min. The time spent in the open arms and track movements of mice were recorded by a video camera system (SMART 3.0; Panlab S. L., Barcelona, Spain). The criterion for entering an open arm was defined as 50% of the body being positioned within the arm.

#### 2.5.3 Tail suspension test

The tail suspension test (TST) was used to assess depression-like behaviors. Briefly, the mice were suspended upside-down by their tail above the floor (from a 60 cm height) using an adhesive tape placed 1 cm from the tip of the tail for 6 min. The time of immobility and motion heat map of mice were recorded using a video camera system (SMART 3.0; Panlab S. L., Barcelona, Spain). The criteria for immobility were defined as complete motionlessness of the body and passive hanging.

#### 2.5.4 Forced swim test

The forced swim test (FST) was used to detect depression-like behaviors in mice. Mice were placed in a clear glass cylinder (45 cm high; 20 cm in diameter) filled with water up to a height of 25 cm (24°C ± 1°C) for 6 min. The time of immobility and motion heat map of mice were recorded using a video camera system (SMART 3.0; Panlab S. L., Barcelona, Spain). The criteria for immobility were defined as complete motionlessness of the body and passive hanging.

#### 2.5.5 Sucrose preference test

The sucrose preference test (SPT) was used to detect anhedonia, a core symptom of depression (Liu et al., 2018). In brief, after being deprived of water for 12 h, mice were placed in separate cages with two bottles for 12 h; one containing 1% sucrose solution (w/v) and the other containing tap water. The positions of bottles were switched after 6 h to avoid possible conditioned place preference. The formula for calculating the sucrose preference rate was as follows: sucrose preference rate (%) = {[sucrose consumption (g)/[sucrose consumption (g) + water consumption (g)]} × 100.

#### 2.5.6 Y-maze test

The Y-maze test (YMT) was used to assess memory deficit, a common and concomitant symptom of depression (Jin et al., 2020). The apparatus used in this experiment had three closed arms (50 × 10 cm, with 40 cm walls) situated 120° apart with a center platform. Each mouse was gently placed on the central platform facing the same maze and was allowed to freely explore the three arms for 6 min. The sequence of entries into the arms was recorded by a video camera system (SMART 3.0; Panlab S. L., Barcelona, Spain). The criterion for entering an arm was defined as 50% of the body being positioned within the arm. Successful alternation was defined as the mouse entering the third arm consecutively before entering the two previously-entered arms. The following calculation was used: spontaneous alternation (%) = (number of successful alternations/total number of arm entries—2) × 100%.

### 2.6 Western blot analysis

After the mice were euthanized, the prefrontal cortex (PFC) and hippocampus (HIP) of each mouse were separated and collected immediately on ice. Then, the total proteins were extracted by homogenization using lysis buffer (RIPA, P0013K, Beyotime, Shanghai, China). Protein extracts were separated by sodium dodecyl sulfate-polyacrylamide gel electrophoresis (SDS-PAGE) and then transferred onto a polyvinylidene fluoride (PVDF) membrane. Then, the PVDF membranes were incubated with the corresponding specific antibodies for NF-κB, COX-2, NLRP3, nuclear factor erythroid 2-related factor (Nrf2), heme oxygenase-1 **(**HO-1), GAPDH, and β-actin overnight at 4°C, followed by incubation with either HRP-labeled Goat Anti-Rabbit IgG (H + L) or HRP-labeled Goat Anti-Mouse IgG (H + L) for 2 h at room temperature. Images of the resulting bands were obtained after ECL (P0018S, Beyotime, Shanghai, China) and developed by the Amersham imager 680 (General Electric Company, Boston, United States). The band intensities were normalized to β-actin or GAPDH. Information on the antibodies utilized in this study is provided in Supplementary Table S3.

### 2.7 Immunofluorescence assay

Coronal cryotome sections (10 μm) were cut from the PFC and HIP and then perfused with PBS and 4% paraformaldehyde. After antigen blocking with QuickBlock™ Blocking Buffer (Beyotime, Shanghai, China) at room temperature for 1 h, the brain sections were incubated with the GFAP, Neun, and IBA-1 antibodies overnight at 4°C, followed by incubation with Alexa Fluor 488-labeled Goat Anti-Mouse IgG (H + L) and Alexa Fluor 488-labeled Donkey Anti-Rabbit IgG (H + L). The nucleus was counterstained with DAPI for 10 min. Images were obtained using a VS120 microscope (OLYMPUS).

### 2.8 ELISA assay

Brain tissue samples were homogenized using RIPA buffer (P0013K, Beyotime, Shanghai, China) and then centrifuged to obtain the supernatant. The levels of IL-1β, IL-6, and TNF-α in the brain were detected by the Mouse IL-1β ELISA Kit (PI301, Beyotime, Shanghai, China), IL-6 ELISA Kit (PI326, Beyotime, Shanghai, China), and TNF-α ELISA Kit (PT512, Beyotime, Shanghai, China), respectively, according to the manufacturer’s protocols. The absorbance was measured at 450 nm using a microplate reader (Multiskan FC, Thermo, Finland).

### 2.9 16S rRNA sequencing and data processing

Fresh fecal samples were collected from the mouse intestine and quickly frozen with liquid nitrogen. DNA was extracted using the PowerSoil DNA Isolation Kit (MO BIO Laboratories, Qiagen N.V.). For 16S rDNA library construction, the V3-V4 region of the bacterial 16S rRNA gene was amplified by the primer pairs 338F (5′-ACT​CCT​ACG​GGA​GGC​AGC​AG-3′) and 806R (5′-GGACTACHVGGGTWTCTAAT-3′) using the thermocycler PCR system (GeneAmp 9,700, ABI, United States). Then, all samples were sequenced on the Illumina Miseq platform (Illumina, Inc., United States) using a Miseq Reagent Kit V3 (600-cycle) (MS-102-3033, Illumina, United States) based on the manufacturer’s instructions.

The resulting sequences were quality filtered using the fastq-join algorithm (Version: 1.3.1) and merged *via* PEAR (Version: 0.9.11). Operational taxonomic unit (OTU) clustering was performed using USEARCH (Version: 11.0.667, http://www.drive5.com/usearch/) with 97% sequence similarity. In order to obtain the species classification information corresponding to each OTU, the RDP Classifier algorithm (with a default confidence threshold of 0.8) was used for the taxonomic analysis of OTU-representative sequences against the Silva 16S rRNA database (Version: 132, http://www.arb-silva.de). The QIIME software (Version: 2020.2) was used to generate an abundance of information at different taxonomic levels.

Alpha diversity was used to reflect the microbial species diversity within a single sample. In this study, Mothur (version 1.30, http:www.mothur.org/) was utilized to calculate microbial species richness (Chao and ACE) and diversity (Shannon and Simpson index). The Wilcoxon rank-sum test was used to compare the difference between two groups.

Beta diversity was used to reflect the diversity of microbial species among different samples. In this study, beta diversity was calculated by the Bray Curtis measure and visualized by Principal Component Analysis (PCA, a technique for data dimensionality reduction) using the R package ‘vegan’.

Linear discriminant analysis (LDA) effect size (LEfSe) was used to identify potential biomarker species with significant differences in abundance between groups. Briefly, the non-parametric Kruskal–Wallis rank-sum test was used to detect microbial species with significant differences in abundance among different groups. Then, the Wilcoxon rank-sum test was used to analyze the differences between groups for species with significant differences obtained in the previous step. Finally, the LDA score was used for data dimensionality reduction and to evaluate the influences of species with significant differences (the default confidence threshold was 4.0). The results were visualized by constructing a histogram of LDA and an evolutionary branching diagram using the R package.

### 2.10 Transmission electron microscopy

Fresh PFC and HIP tissues (1 mm × 1 mm × 1 mm) were obtained and placed on ice immediately after the mice were euthanized. The tissue was fixed with glutaraldehyde for 4 h, then 1% osmium buffer for 2 h, and finally dehydrated with gradient alcohol precipitation. After immersion in acetone/epoxy resin (2:1), acetone/epoxy resin (1:1), and epoxy resin solution, the tissue was then embedded in epoxy resin. The hypothalamus sample was cut into slices approximately 50 nm thick and then soaked in uranium dioxane acetate for 45 min to prepare the sample for observation under electron microscopy. Neuronal ultrastructure images were collected by the H-7650 transmission electron microscope.

### 2.11 Statistical analyses

All data in this study were measured by an independent investigator who was blinded to the experimental conditions. The data were analyzed using SPSS (Version 22.0, SPSS, Inc.; Chicago, IL, United States). The difference between two groups was analyzed using the Student’s t-test (Control group vs*.* CUS group, Sal group vs*.* CS-150), whereas one-ANOVA followed by Dunnett’s test was adopted to compare several groups (Sal group vs*.* CS-50, CS-100, and CS-150 group). Pearson correlation analysis was used to analyze the correlation between the two indexes. Statistical analysis of the 16S rRNA sequence data has been described in Section 2.8. *p* < 0.05 was considered to indicate a significant difference. All data are expressed as the mean ± standard error using GraphPad Prism 8.0.

## 3 Results

### 3.1 CS relieved depression-like behaviors in mice exposed to CUS

In the toxicity test, the mice did not show death and abnormal behavior. So, the LD50 value of CS was concluded to be greater than 1.5 g/kg in mice, indicating CS had almost no toxic effects in this study.

Chronic unpredictable stress is widely used experimentally to induce robust depression-like behavior (Hill et al., 2012). In this study, mice exposed to CUS exhibited anxiety-like behaviors, including spending reduced time in the center of the OFT (t = 3.114, *p* = 0.008, Figures 1B,C) and decreased time in the open arm of the EPMT (*t* = 3.206, *p* = 0.006, Figures 1D,E). After CS treatment at a dose of either 100 or 150 mg/kg, the CUS-induced decrease in the time spent in the central area of the OFT was reversed (ANOVA: F = 6.235, *p* = 0.003; Sal vs*.* CS-100: Dunnett’s test: *p* = 0.007; Sal vs*.* CS-150: Dunnett’s test: *p* = 0.001; Figures 1B,C). When compared to the Sal group, the time spent in the open arms was significantly increased in the CS-150 group (ANOVA: F = 5.971, *p* = 0.004; Sal vs*.* CS-100: Dunnett’s test: *p* = 0.002; Figures 1D,E). Although the CS-50 and CS-150 groups each showed a tendency for increased time spent in the open arms, there was no difference when compared to the Sal group (ANOVA: F = 5.971, *p* = 0.004; Sal vs*.* CS-50: Dunnett’s test: *p* = 0.687; Sal vs*.* CS-150: Dunnett’s test: *p* = 0.069; Figures 1D,E). Taken together, CS could relieve anxiety-like behaviors in mice exposed to CUS.

The immobility time in the TST (*t* = 3.184, *p* = 0.007, Figures 1F,G) and FST (*t* = 5.878, *p* < 0.001, Figure 1H) was significantly prolonged in the CUS group when compared to the Control group, which suggests that mice exhibited significant depression-like behaviors after exposure to CUS. Further, the immobility time of mice in the CS-150 group was significantly shortened in both the TST (ANOVA: F = 5.710, *p* = 0.004; Sal vs CS-150: Dunnett’s test: *p* = 0.005; Figures 1F,G) and FST (ANOVA: F = 5.971, *p* = 0.004; Sal vs CS-150: Dunnett’s test: *p* < 0.001; Figure 1H). Additionally, the sucrose preference rate was significantly reduced after exposure to CUS (*t* = 5.878, *p* < 0.001, Figure 1I), but this effect was reversed by CS administration (ANOVA: F = 5.732, *p* = 0.004; Sal vs*.* CS-50: Dunnett’s test: *p* = 0.010; Sal vs*.* CS-100: Dunnett’s test: *p* = 0.026; Sal vs*.* CS-150: Dunnett’s test: *p* = 0.002; Figure 1I). In conclusion, CS could relieve depression-like behaviors in mice exposed to CUS.

Although memory deficits are a concomitant symptom of depression, the mice included in our study did not show impaired memory after exposure to CUS (*t* = 1.307, *p* = 0.212, Figure 1J). Meanwhile, after CS administration, spontaneous alternation in all CS groups showed no significant change when compared to the Sal group (ANOVA: F = 0.331, *p* = 0.803; Figure 1J).

### 3.2 CS alleviated CUS-induced neuroinflammation in mice

**FIGURE 2:**
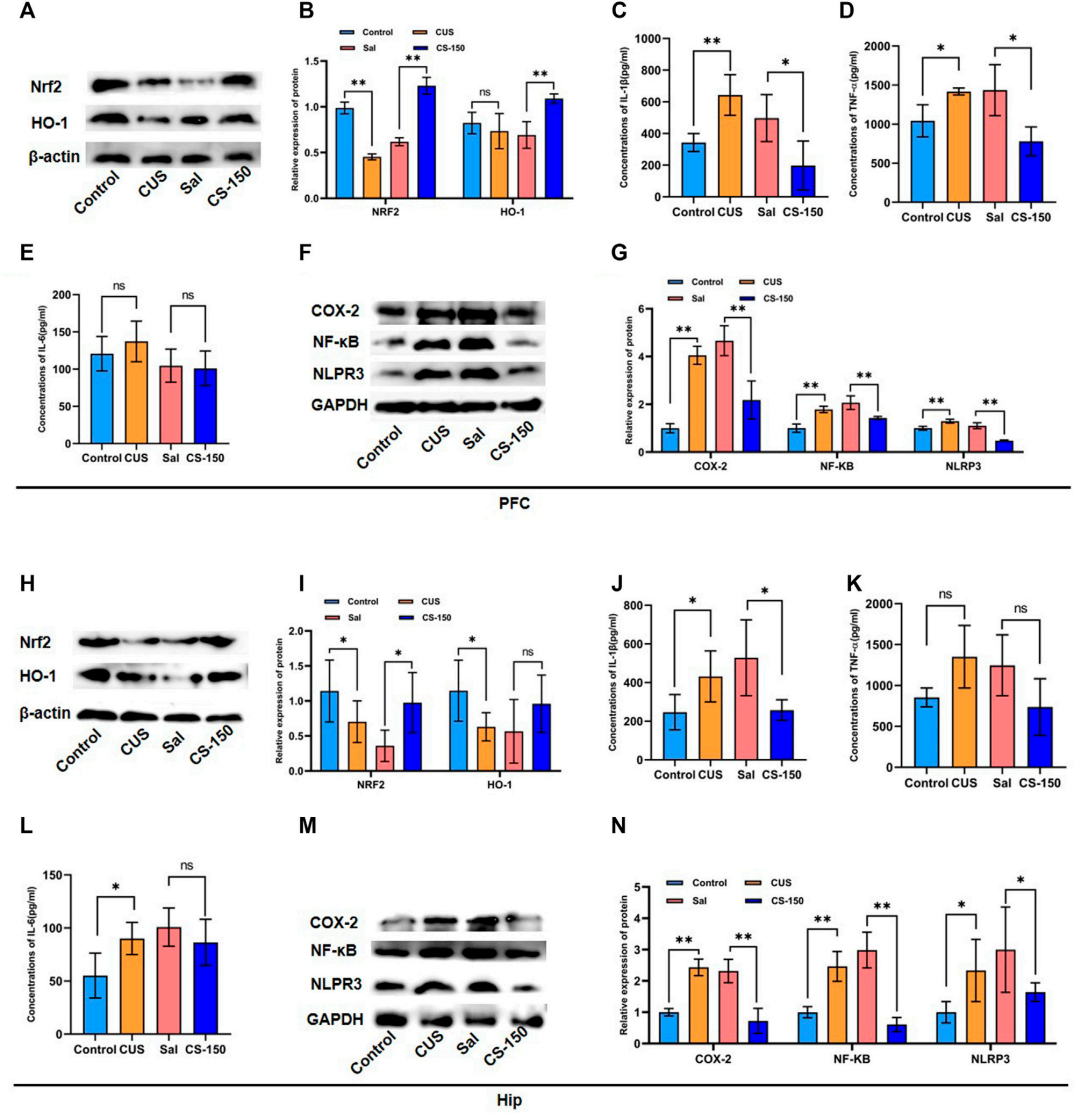
CS alleviated CUS-induced neuroinflammation in both the PFC and HIP. **(A, B)** Protein bands and relative expression levels of NRF2 and HO-1 in the PFC obtained by Western blot. **(C–E)** Concentrations of IL-1β, TNF-α, and IL-6 in the PFC measured by the ELISA assay. **(F, G)** Protein bands and relative expression levels of NLRP3, COX-2, and NF-κB in the PFC obtained by Western blot. **(H, I)** Protein bands and relative expression levels of NRF2 and HO-1 in the PFC obtained by Western blot. **(J–L)** Concentrations of IL-1β, TNF-α, and IL-6 in the PFC measured by the ELISA assay. **(M, N)** Protein bands and relative expression levels of NLRP3, COX-2, and NF-κB in the PFC obtained by Western blot. Data are expressed as the mean ± standard error. *p < 0.05, **p < 0.01.

The HIP and PFC have been repeatedly implicated in the pathophysiology and progression of depression. And animal models have demonstrated that chronic unpredictable stress promotes the production of pro-inflammatory cytokines in the hippocampus and mPFC (Belleau et al., 2019). So, two important brain regions, PFC and HIP, were selected as target brain regions in the study to explore the effect of Cuscutae Semen on improving inflammation in depression. Meanwhile, neuroinflammation plays an important role in the pathogenesis of depression, and continuous oxidative stress can cause inflammation (Ahmed et al., 2017). Hence, we first detected Nrf2 and HO-1 protein expression levels in the mouse brain. The results showed that the expression levels of Nrf2 proteins were decreased in both the PFC (*t* = 18.318, *p* < 0.001, Figures 2A,B) and HIP (*t* = 2.016, *p* = 0.041, Figures 2H,I) after exposure to CUS. These deficits were rescued by CS at a dose of 150 mg/kg (PFC: *t* = 14.798, *p* < 0.001, Figures 2A,B; HIP: *t* = 3.111, *p* = 0.011; Figures 2H,I). As shown in Figures 2B,I, following exposure to CUS, the expression of HO-1 protein was downregulated in the HIP (*t* = 2.627, *p* = 0.025, Figure 2I), but not in PFC (*t* = 0.971, *p* = 0.354, Figure 2B). CS elevated the protein level of HO-1 in the PFC (*t* = 6.320, *p* < 0.001, Figure 2B), but this phenomenon was not observed in the HIP (*t* = 1.576, *p* = 0.146, Figure 2I).

To explore the effect of CS on neuroinflammation, several proinflammatory factors were measured, including IL-1β, TNF-α, and IL-6. As shown in Figures 2C–E, the levels of IL-1β (PFC: *t* = 4.280, *p* = 0.005, Figure 2C) and TNF-α (PFC: *t* = 3.023, *p* = 0.039, Figure 2D) were upregulated in mice exposed to CUS, and this effect was rescued by CS treatment (IL-1β: *t* = 2.799, *p* = 0.031, Figure 2C; TNF-α: *t* = 3.111, *p* = 0.011; Figure 2D). Meanwhile, after CUS exposure and CS administration, the expression levels of IL-1β exhibited similar changes in the HIP of mice (Figure 2J). But there was no significant change in TNF after CUS and CS administration (Figure 2K). However, there was no significant difference in the IL-6 expression between the Control group and CUS group in the PFC of mice (*t* = 0.924, *p* = 0.391, Figure 2E), but not in the HIP (*t* = 2.685, *p* = 0.036, Figure 2L). Notably, after CS treatment, IL-6 levels showed no significant changes in either the PFC (*t* = 0.230, *p* = 0.826, Figure 2E) or the HIP (*t* = 1.016, *p* = 0.349, Figure 2L). We further detected inflammatory proteins, including NF-κB, COX-2, and NLRP3. The results in Figures 2F,G show that the protein levels of NF-κB (*t* = 8.780, *p* < 0.001), COX-2 (*t* = 17.538, *p* < 0.001), and NLRP3 (*t* = 6.352, *p* < 0.001) were upregulated in the CUS group when compared to the Control group. This tendency for elevation in the protein levels of NF-κB (*t* = 5.994, *p* < 0.001), COX-2 (*t* = 5.359, *p* < 0.001), and NLRP3 (*t* = 12.138, *p* < 0.001) was reversed by CS administration. Moreover, this same trend for NF-κB, COX-2, and NLRP3 protein was observed in the HIP after exposure to CUS followed by CS treatment (*p* < 0.05, Figures 2M, N).

### 3.3 CS decreased CUS-induced microglial and astrocyte activation

**FIGURE 3:**
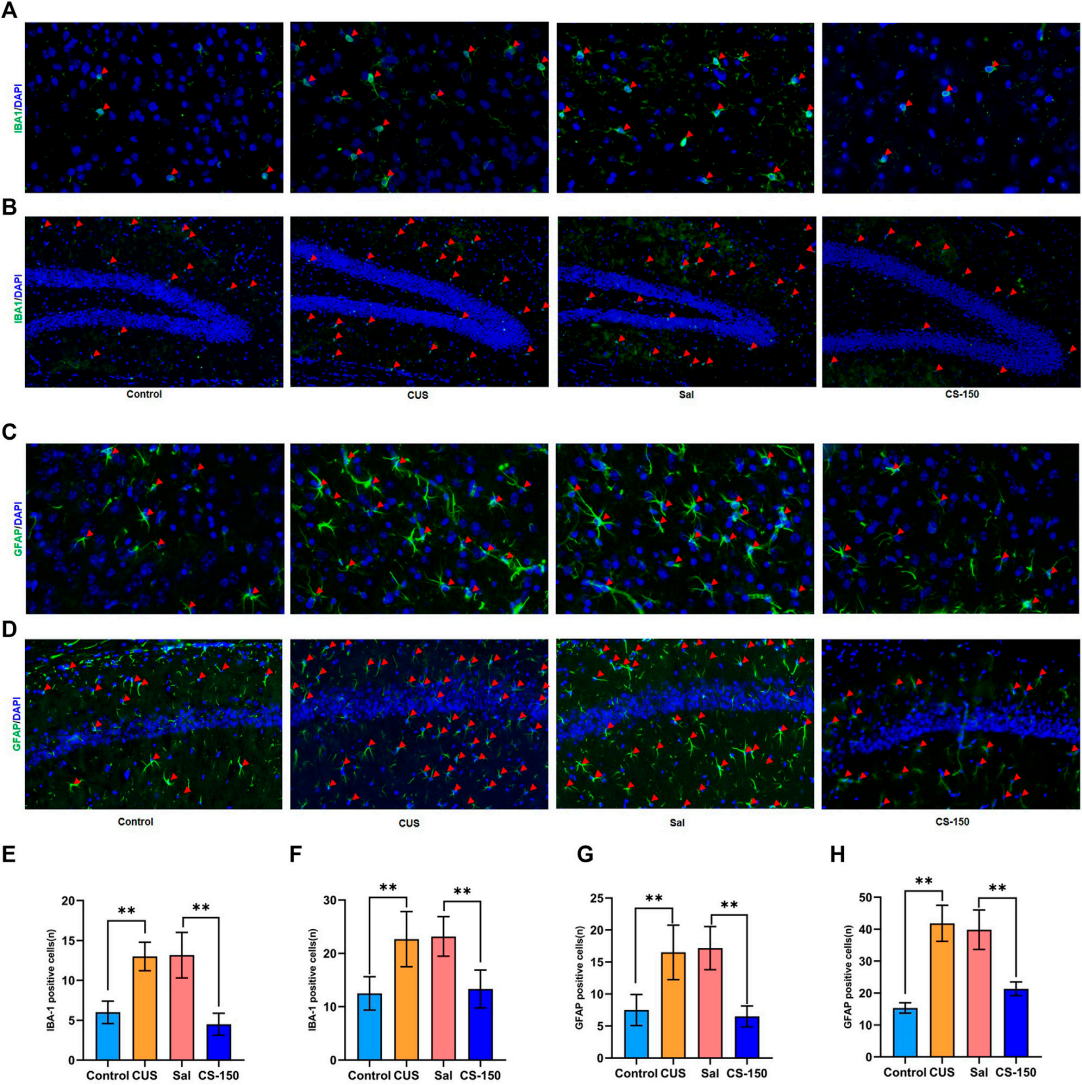
CS decreased CUS-induced microglial and astrocyte activation in both the PFC and HIP. **(A, B)** Images of IBA1-positive cells in the PFC (under ×400 magnification) and DG (under ×200 magnification) obtained by immunofluorescence. **(C, D)** Images of GFAP-positive cells in the PFC (under ×400 magnification) and CA1 (under ×200 magnification) obtained by immunofluorescence. **(E, F)** The number of IBA1-positive cells. **(G, H)** The number of IBA1-positive cells. Data are expressed as the mean ± standard error. *p < 0.05, **p < 0.01, and *p <* 0.05.

Activated glial cells, including microglia and astrocytes, are key sources of neuroinflammation and central cytokines (Kwon and Koh, 2020). Therefore, the immunofluorescence assay was performed to detect the microglial activation marker IBA1 and the astrocyte activation marker GFAP. As shown in Figure 3, the number of cells positive for IBA1 was increased in the CUS group in both the PFC (*t* = 7.519, *p* < 0.001, Figures 3A,E) and dentate gyrus (DG) (*t* = 4.118, *p* = 0.002, Figures 3B,F). CS reversed this increase in the number of IBA1-positive cells in both the PFC (*t* = 6.691, *p* < 0.001, Figures 3A,E) and DG (*t* = 4.685, *p* = 0.001, Figures 3B,F). However, the number of IBA1-positive cells in CA1 did not change significantly in any group (Supplementary Figure S2). We further investigated the effect of CS on astrocytes in the PFC and HIP. The number of GFAP-positive cells was upregulated in both the PFC (*t* = 4.519, *p* = 0.001, Figures 3C,G) and CA1 (*t* = 11.062, *p* < 0.001, Figures 3D,H) after exposure to CUS, while CS reversed this increase in the number of GFAP-positive cells in both the PFC (*t* = 6.966, *p* < 0.001, Figures 3C,G) and CA1 (*t* = 6.924, *p* < 0.001, Figures 3D,H). However, the number of GFAP-positive cells in the DG showed no significant change in any group (Supplementary Figure S3). These results indicate that CS decreased CUS-induced microglial and astrocyte activation in both the PFC and HIP.

### 3.4 CS altered the structure of gut microbiota in mice exposed to CUS

**FIGURE 4:**
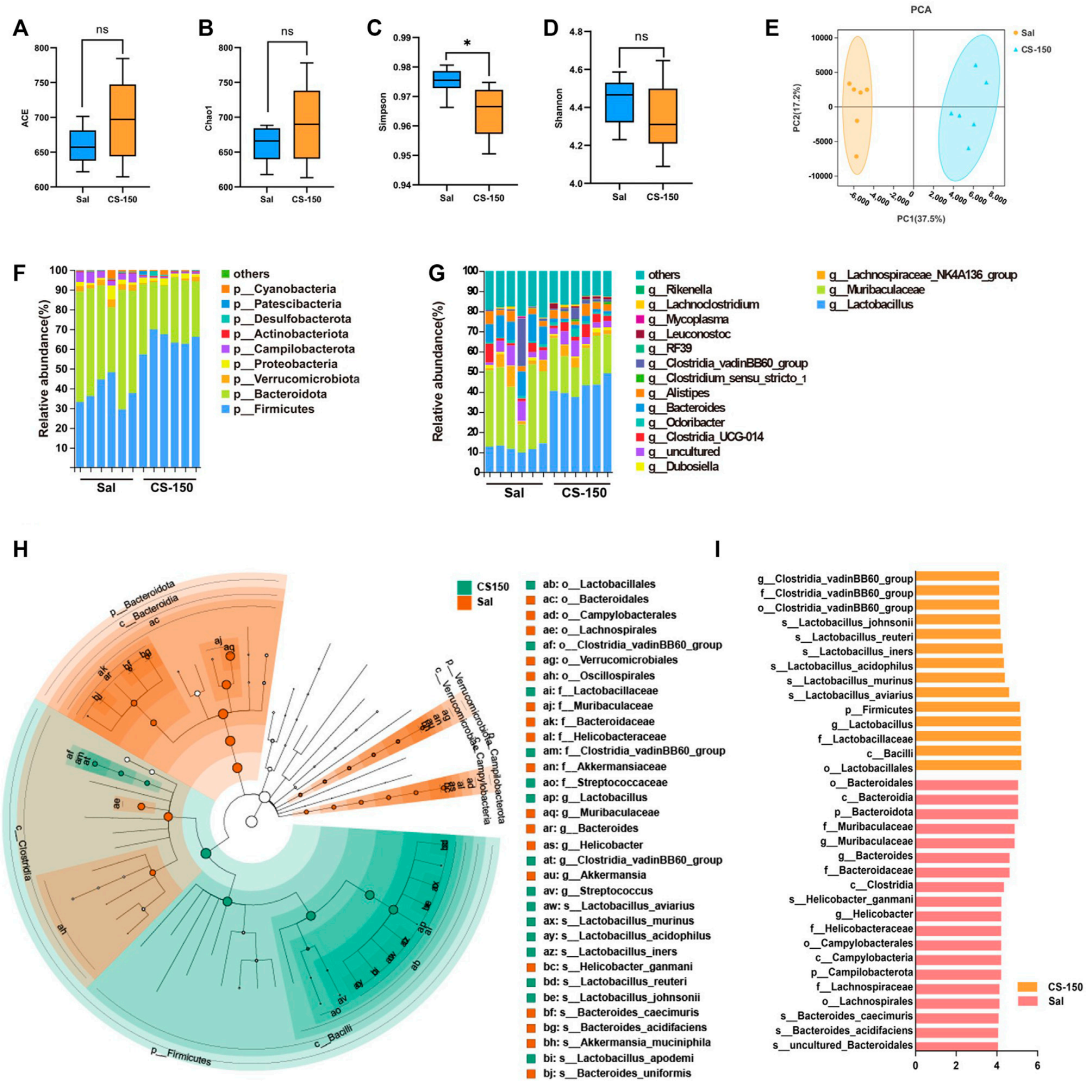
CS altered gut microbiota structure in CUS-exposed mice. **(A–D)** The ACE, Chao1, Simpson, and Shannon index values of the Sal group and CS-150 group. **(E)** PCA based on the Bray-Curtis distance for the gut microbiota. **(F)** Microbial distribution at the phylum level. **(G)** Microbial distribution at the genus level. **(H, I)** Cladogram and histogram of LDA scores greater than four based on LEfSe. Data are expressed as the mean ± standard error. *p* < 0.05 indicates a statistically significant difference.

To explore the changes in the gut microbiome of mice exposed to CUS under CS treatment, 16S rRNA sequencing was performed. As shown in Figure 4, compared to the Sal group, the ACE (*p* > 0.05, Figure 4A), Chao1 (*p* > 0.05, Figure 4B), and Shannon (*p* > 0.05, Figure 4D) index values of the CS treatment groups did not change, while the Simpson index value of the CS-150 group was significantly decreased compared to that of the Sal group (*p* < 0.05, Figure 4C). The results of PCA showed that there was a significant clustering difference between the CS-150 and Sal groups (Figure 4E). The dominant taxa in the mouse gut microbiota were determined to be *Firmicutes* and *Bacteroidota*. Mice treated with CS consistently exhibited an increased abundance of *Firmicutes* (*p* < 0.05, Figure 4F). Further analysis of the effects of CS on the intestinal flora abundance at the genus level showed that the relative abundance of *Lactobacillus* was significantly increased in the CS-150 group when compared to the Sal group (*p* < 0.05, Figure 4G), while the relative abundance of Muribaculaceae *was* significantly decreased (*p* < 0.05, Figure 4G). LEfSe was employed to identify potential biomarker species with significant differences in abundance, and the results showed that the dominant taxa in the CS group were *Firmicutes* at the phylum level, *Bacilli* at the class level, Lactobacillales at the order level, L*actobacillaceae* at the family level, *Lactobacillus* at the genus level, and *Lactobacillus aviarius* at the species level (LDA >4, *p* < 0.05, Figures 4H,I). These data indicate that CS changed the structure of the gut microbiota and increased the relative abundance of *Lactobacillus*.

### 3.5 Behaviors and neuroinflammation-related indicators correlated with *lactobacillus* species

**FIGURE 5:**
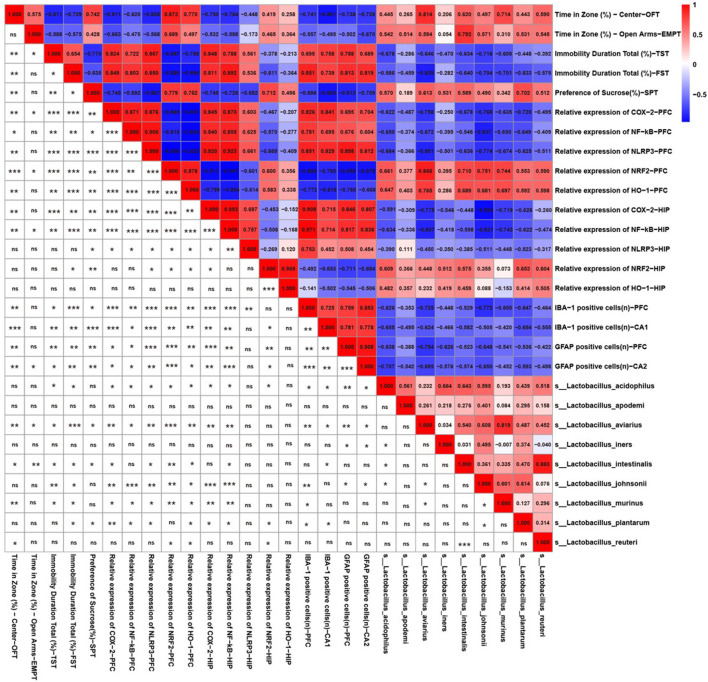
Behavior and neuroinflammation-related indicators positively/negatively correlated with the relative abundance of *Lactobacillus* species. Spearman correlations among *Lactobacillus* species, depression-like behaviors, and neuroinflammation-related indicators. Numbers in the upper right area represent values of the correlation coefficient; symbols in the lower left area represent the results of the correlation and significance test. *p < 0.05, **p < 0.01, ***p < 0.001.

Because CS significantly increased the relative abundance of *Lactobacillus* according to the 16s rRNA sequencing results, we further identified the correlations between behavior, neuroinflammation-related indicators, and *Lactobacillus* species. As shown in Figure 5, there was a significant relationship between the abundances of *L. aviarius* and *Lactobacillus intestinalis* and behavioral indicators. *Lactobacillus acidophilus*, *L. aviarius*, *Lactobacillus johnsonii*, *and Lactobacillus murinus* were positively correlated with markers of oxidative stress, including NRF2 and HO-1, but were negatively correlated with inflammatory proteins, including NF-κB, COX-2, and NLRP3. Meanwhile, there was a negative relationship between the abundances of *L. aviarius* and *L. acidophilus* and IBA1-and GFAP-positive cells. However, *Lactobacillus apodemi* did not show any correlation with either behavioral or neuroinflammation-related indicators. This indicates that *Lactobacillus* is a mediator of CS in alleviating depression-like behaviors and neuroinflammation in mice.

### 3.6 CS changed the synaptic ultrastructure

**FIGURE 6:**
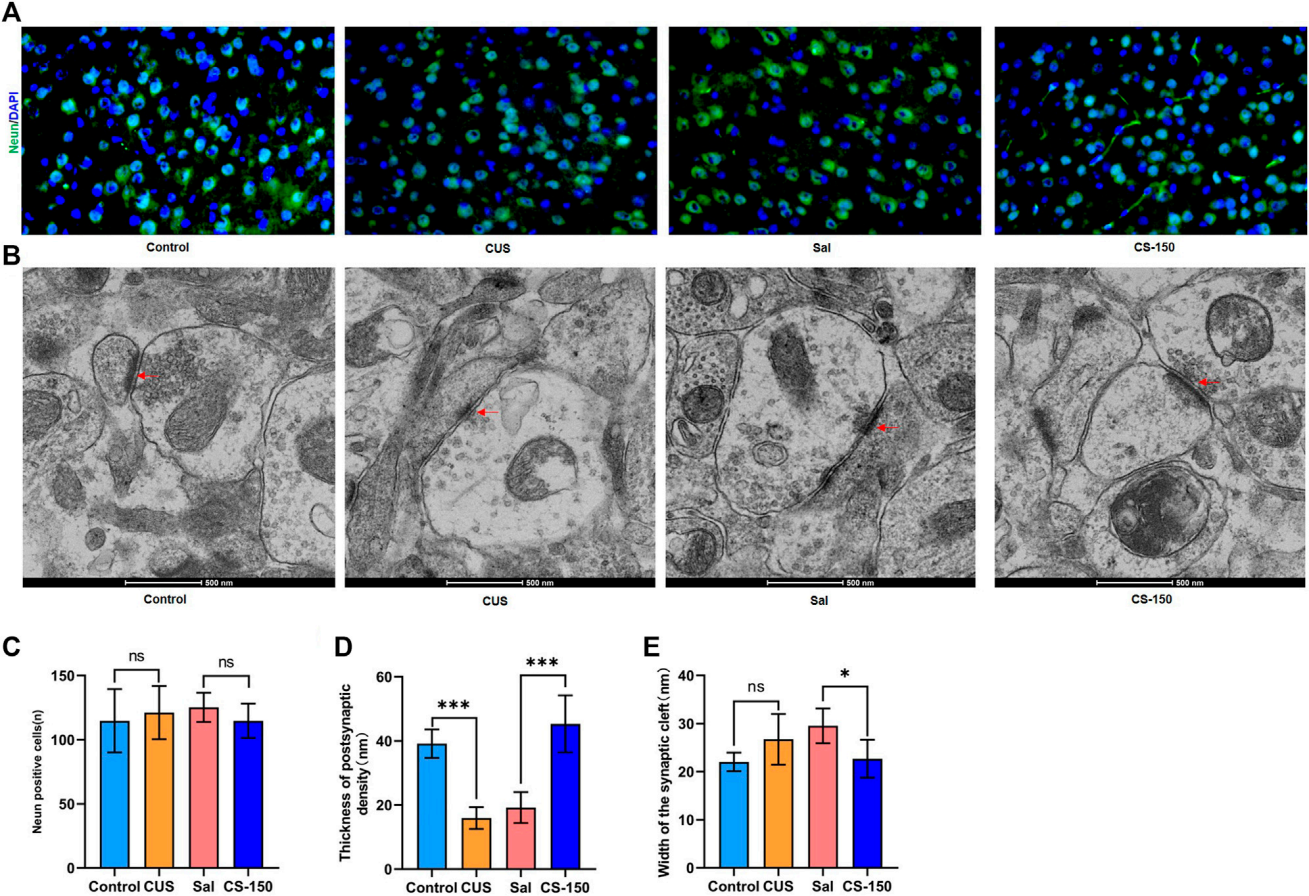
CS altered the synaptic ultrastructure. **(A)** Images of Neun-positive cells in the PFC (under ×400 magnification) obtained by immunofluorescence. **(B)** Synaptic ultrastructure of the mPFC under ×60,000 magnification. **(C)** The number of Neun-positive cells. **(D)** Thickness of PSD of the PFC. **(E)** Synaptic cleft width of the PFC. Data are expressed as the mean ± standard error. *p < 0.05, **p < 0.01.

There was no significant change found in the number of Neun-positive cells after exposure to CUS (*t* = 0.494, *p* = 0.632, Figures 6A,C), and CS administration did not significantly affect the number of Neun-positive cells in the PFC (*t* = 1.464, *p* = 0.174, Figures 6A,C). Meanwhile, the number of Neun-positive cells in both the DG (Supplementary Figure S4) and CA1 (Supplementary Figure S5) exhibited a similar phenomenon. Transmission electron microscopy showed that postsynaptic density in the PFC was downregulated in the CUS group (*t* = 10.110, *p* < 0.001, Figures 6B,D), while it was upregulated in the CS-150 group (*t* = 6.318, *p* < 0.001, Figures 6B,D). Although there was no change in the width of the synaptic cleft in the PFC of the CUS group when compared to that of the control group (*t* = 2.060, *p* = 0.066, Figures 6B,E), there was a significant reduction in the synaptic cleft of the CS group when compared to that of the Sal group (*t* = 3.149, *p* = 0.010, Figures 6B,E). There was no narrowing of the synaptic cleft after CS treatment in the HIP (Supplementary Figures S6A, S6C), however, changes in postsynaptic density in the HIP were similar to those observed in the PFC (Supplementary Figures S6A, S6B). These results suggest that CS altered the synaptic ultrastructure, but did not affect the number of neurons in mice.

## 4 Discussion

The gut microbiota-neuroinflammation axis is involved in the pathophysiology of MDD (Inserra et al., 2018; Rutsch et al., 2020), and is hence considered a novel pharmacological target for the treatment of the disease (Misra and Mohanty, 2019). In this study, we found that CS could relieve depression-like behaviors and neuroinflammation induced by CUS. The CS-induced suppression of over-activated microglia and astrocytes changed the structure of the mouse gut microbiota and increased the relative abundance of *Lactobacillus*. Meanwhile, the relative/absolute abundance of *Lactobacillus* was positively/negatively correlated with behavioral- and inflammation-related indexes. Moreover, CS could reverse the synaptic structural defects caused by CUS. These results suggest that CS alleviates CUS-induced depression-like behaviors in mice *via* regulating the gut microbiota-neuroinflammation axis.

The CUS protocol is commonly used to create a robust animal model of depression that manifests a similar phenotype to that caused by environmental factors in humans with depression (Hill et al., 2012). Several studies have reported that CUS could induce anxiety-like behaviors in mice (Li et al., 2019a; Liu et al., 2021). In this study, after exposure to CUS, the mice showed anxiety-like behaviors, including a reduction in time spent in both the center of the OFT and in the open arms of the EPMT. Meanwhile, the immobility times of mice were reduced in both the FST and TST following CUS exposure, which is consistent with a previous report (Leng et al., 2018). Moreover, anhedonic behavior, which is considered the main symptom of depression (Antoniuk et al., 2019), was also induced in the SPT by the CUS protocol in this study. These results jointly suggest that we successfully replicated a mouse model of depression based on the CUS protocol. Although impaired memory is a characteristic feature of major depression (Porter et al., 2007), there was no significant change observed in the spontaneous alternation of mice exposed to CUS in this study. We speculate that the main reason for this result is that CUS may have greater heterogeneity in inducing impairments in working memory. After treatment with CS at a dose of either 50, 100, or 150 mg/kg, the depression-like behaviors of mice observed in the behavioral tests was ameliorated to varying degrees in a dose-dependent manner. Among the doses, 150 mg/kg of CS exhibited the strongest antidepressant effect. It is worth noting that quercitrin (Sun et al., 2021), isoquercitrin (Martinez-Hernandez et al., 2021), hypericin (Zhai et al., 2022), chlorogenic acid (Lim et al., 2018), kaempferol (Li et al., 2022) and astragalin (Yao et al., 2022) identified in CS extracts have all been previously reported to have antidepressant effects. Among them, hypericin is also the main component of the traditional antidepressant St. John’s Wort, which has shown good antidepressant effects in randomized controlled clinical trials (Linde et al., 2008). And Song reported that chlorogenic acid significantly alleviates adrenocorticotropic hormone-induced depression-like behavior, as demonstrated by a significant increase in sucrose preference and a significant increase in immobility time for TST (47). Taken together, this study found that CS could relieve depression-like behaviors in mice exposed to CUS, which provides new insight into the limitations of antidepressants and the potential of other therapeutic agents.

Neuroinflammation, a central process in the inflammatory hypothesis of depression, mediates increased stress responsiveness and depression susceptibility. At present, the most consistent conclusion regarding the relationship between neuroinflammation and depression is that levels of proinflammatory cytokines; including TNF-α, IL-1β, and IL-6; are increased in both the plasma and central nervous system in patients suffering from MDD (Young et al., 2014). Meanwhile, patients suffering from MDD who had attempted suicide have been found to have higher levels of pro-inflammatory cytokines, which suggests that proinflammatory cytokines influence the progression and severity of depressive disorders (Serafini et al., 2013). In this study, we found that the levels of IL-1β and TNF-α were decreased after CS administration, and that CS could alleviate the CUS-induced expression of these proinflammatory cytokines in the brain. Seok et al. also found that CS extract is capable of suppressing the inflammatory response in LPS-stimulated BV-2 microglia (Kang et al., 2014). Hypericin, one of the rich compounds in CS extract, has been reported to be able to alleviate the symptoms of *postpartum* depression as effective as fluoxetine by decreasing the levels of inflammatory factors (IL-6, IL-1β, TNF - α) in serum (Zhai et al., 2022). And chlorogenic acid, another compound rich in CS extract, was found to relieve the inflammatory stress of LPS-Induced BV2 Cell *via* reducing the production of inflammatory mediators, including IL-6, IL-1β and TNF-α (Xu et al., 2022). IL-6 is one of the few cytokines that can act as both a pro-inflammatory and anti-inflammatory cytokine (Hunter and Jones, 2015). However, in the present study, the level of IL-6 showed no significant change after CUS exposure and CS treatment. Notably, previous research found that the expression data of IL-6 obtained from a cohort of depressed patients exhibited a bipolar distribution pattern (Chocano-Bedoya et al., 2014), and there have been conflicting conclusions regarding IL-6 changes following antidepressant drug treatments (Kenis and Maes, 2002). This discrepancy may be due to the dimorphism of the biological characteristics of IL-6, which needs to be explored further.

The NLRP3 inflammasome, which is a key component of the NLR family that triggers the innate immune response, has been reported to be involved in the pathophysiology and treatment of depression (Kaufmann et al., 2017). In this study, we found that NLRP3 was upregulated in mice exposed to CUS, and that this defect could be reversed by CS. Several studies have also found that restraining the NLRP3 inflammasome attenuated lipopolysaccharide-induced acute depression-like behaviors (Xu et al., 2016; Arioz et al., 2019). Thus, the NLRP3 inflammasome may mediate the effects of drugs that alleviate depression-like behaviors. According to the relevant literature, NLRP3 inflammasome activation generally occurs through two pathways. One is through stimulation of the NF-κB pathway; this activates the translocation of NF-κB into the nucleus, the induction of pro-IL-1β, and the increased synthesis of NLRP3, thereby initiating inflammation (Afonina et al., 2017). The other pathway is through oxidative stress signaling, whereby NRF2, which is a key transcription factor of the cellular response to oxidative stress, negatively regulates NLRP3 inflammasome activity (Ahmed et al., 2017). In this study, NF-κB was downregulated by CS treatment, whereas NRF2 was upregulated by CS treatment in CUS-exposed mice. This indicates that both NRF2 and NF-κB are involved in the inhibitory effect of CS against NLRP3 in the CUS model. Changes in the levels of COX-2, a downstream target protein of NF-κB, were also found to mirror those of NF-κB in this study. According to literature reports, chlorogenic acid exposure in lipopolysaccharide/adenosine triphosphate-stimulated cells leads to a decrease in p-NF-κB and NLRP3 inflammasome-related proteins (Zeng et al., 2020). And chlorogenic acid treatment also promoted the nuclear translocation of NRF2, suppressed oxidative stress, and thus inhibit the expression of NLRP3 (Huang et al., 2022). Moreover, we found that the number of astrocytes and microglia were increased after CUS exposure, whereas they were decreased after CS treatment. It is known that when cells are stressed, these cells are activated, which then enhances the neuroinflammatory response and secretion of pro-inflammatory cytokines, such as TNF-α and IL-1β (Kwon and Koh, 2020). These results reveal that CS could alleviate CUS-induced neuroinflammation in mice.

Dysbiosis of the gut microbiota is reportedly involved in the neurobehavioral changes and pathogenesis of MDD (Bear et al., 2020). The absence of probiotic bacteria in the gut is implicated in susceptibility to depression (Kohler et al., 2017; Owen and Corfe, 2017). The intake of probiotics has been observed to exert favorable effects on anxiety- and depression-related behaviors in both animal and human subjects (Messaoudi et al., 2011). In this study, we found that CS could change the structure of the gut microbiota and increase the relative abundance of *Lactobacillus*. And Song also reports that Chlorogenic acid can alter intestinal microbial community structure, including changes in the relative abundance of key bacteria such as *Vibrio* desulphurica, *Vibrio* desulphurica, *Klebsiella*, Burkholella, and Bifidobacterium, which may contribute to its antidepressant effect (Song et al., 2019). Meanwhile, a significant relationship was also observed between several Lactobacilli and indicators of depression-like behaviors. This finding is consistent with Rudzki’s research, which showed that augmentation with *Lactobacillus* decreased depressive symptoms and improved cognitive function in patients suffering from MDD in a randomized controlled trial. One study also reported that *Bifidobacterium* exerted positive antidepressant effects (Tian et al., 2022). However, in the current study, there was no significant change found in the relative abundance of *Bifidobacterium* after CS treatment. The neuroinflammation signal might be one of the pathways by which the gut microbiota affects depression (Carlessi et al., 2021). The NLRP3 inflammasome is considered to be an immune sentinel for sensing gut bacteria, and has been shown to be the hub of both gut microbiota and the neuroimmune system (Pellegrini et al., 2020). In this study, we found that the majority of the *Lactobacillus* species correlated with the oxidative stress protein NRF2 and several inflammation-related indicators, including NF-κB, COX-2, and NLRP3. Accumulating evidence suggests that antidepressants alleviate depression-like behaviors and neuroinflammation by regulating gut bacteria (Li et al., 2019b; Cao et al., 2020). Taken together, CS appears to alleviate CUS-induced depression-like behaviors in mice *via* regulating the gut microbiota-neuroinflammation axis.

Synaptic structures can respond adaptively to changing environments, such that the structural remodeling of neurons occurs after stress; this process is involved in depression and cognitive deficits (Shen et al., 2019). In the present study, we observed a decrease in the PSD after exposure to CUS, and this was alleviated after treatment with CS, which indicates that CS can remodel the synaptic ultrastructure. A relevant study showed that neuroinflammation induced lipopolysaccharide-associated anxiety- and depressive-like behaviors *via* modulating neuronal plasticity (Zheng et al., 2021). Overactivated astrocytes also affect synaptic plasticity by enveloping the extracellular matrix around synapses (Nguyen et al., 2020; Liu et al., 2022). Talani et al. found that treatment with *Bifidobacterium* improved hippocampal plasticity by increasing dendritic spine density (Talani et al., 2020). Thus, combined with their results, we hypothesize that CS mediates the synaptic structure through the gut microbiota-neuroinflammation axis in the CUS model.

This work proved that CS alleviates CUS-induced depression-like behaviors in mice *via* regulating the gut microbiota-neuroinflammation axis. Chlorogenic acid and hypericin, detected in abundance in CS extracts, were shown to have significant antidepressant and anti-inflammatory effects, which may be the main active substances in which CS functions. Therefore, the combination of multiple active components in CS extract is the key to realize the antidepressant like behavior. The multi-component and multi-target mode of action is the advantage of CS extract to exert the antidepressant effect, and also provides a new idea for the development of antidepressant drugs. In summary, this present study suggests that CS has potential value as an alternative medicine for treating MDD, and provides a new mechanistic theory by which CS regulates the gut microbiota-neuroinflammation axis.

## Data Availability

The raw data supporting the conclusions of this article will be made available by the authors, without undue reservation.
