# Supplementary material for: Cuscutae semen alleviates CUS-induced depression-like behaviors in mice via the gut microbiota-neuroinflammation axis
Source: Front Pharmacol. 2023 Feb 24;14:1107781. doi: 10.3389/fphar.2023.1107781 (PMC9998491; doi:10.3389/fphar.2023.1107781)
Supplement: Supplementary file 1 [file DataSheet1.PDF]

Supplementary Figure 1

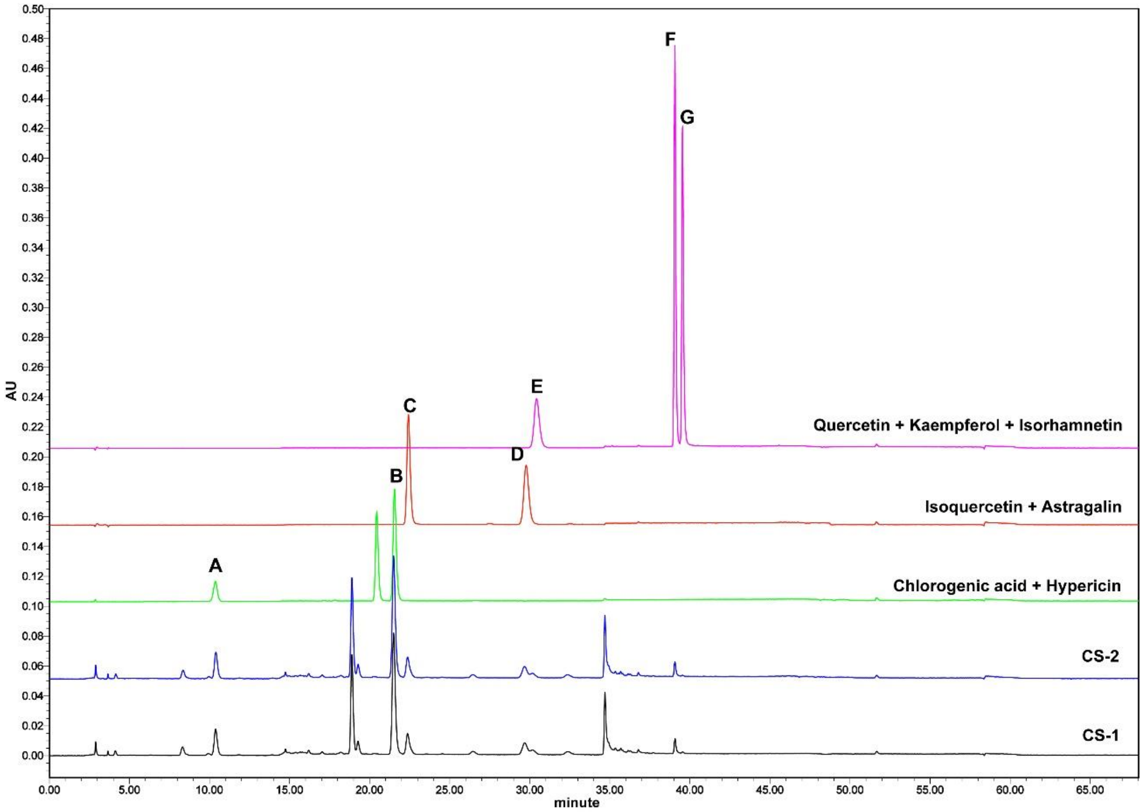

Supplementary Figure 1. Chromatograms of HPLC analysis. CS and seven standards (quercetin, isoquercetin, astragalin, hypericin, kaempferol, chlorogenic acid, and isorhamnetin) were analyzed by using a Shimadzu HPLC system (Waters 2695). The chromatographic conditions: column: (PFchrom EP C18, 4.6*250mm, 5um); detection wavelength: 200 nm-600 nm; injection volume: 10ul; flow rate: 1.0ml/min; and run time: 65 min. A: Chlorogenic acid; B: Hypericin; C: Isoquercetin; D: Astragalin; E: Quercetin; F: Kaempferol; G: Isorhamnetin.

Supplementary Figure 2

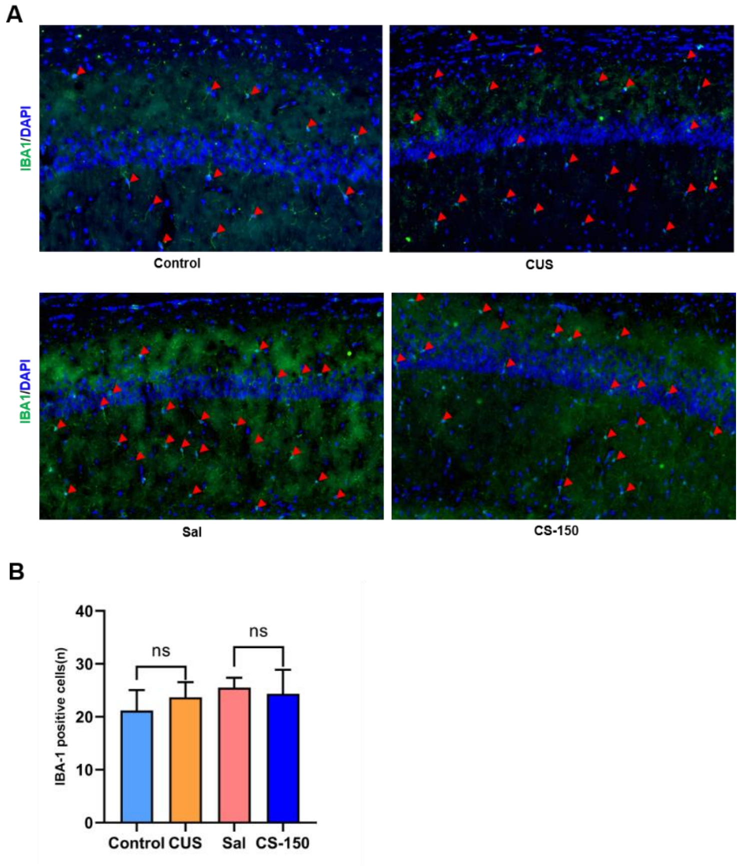

Supplementary Figure 2. CS had no effect on microglial in CA1. (A) Images of IBA1 positive cell in CA1 (under ×200 magnification) obtained from the immunofluorescence test. (B) The number of IBA1 positive cell. The data are expressed as the mean ± standard error. ns $P > 0.05$.

Supplementary Figure 3

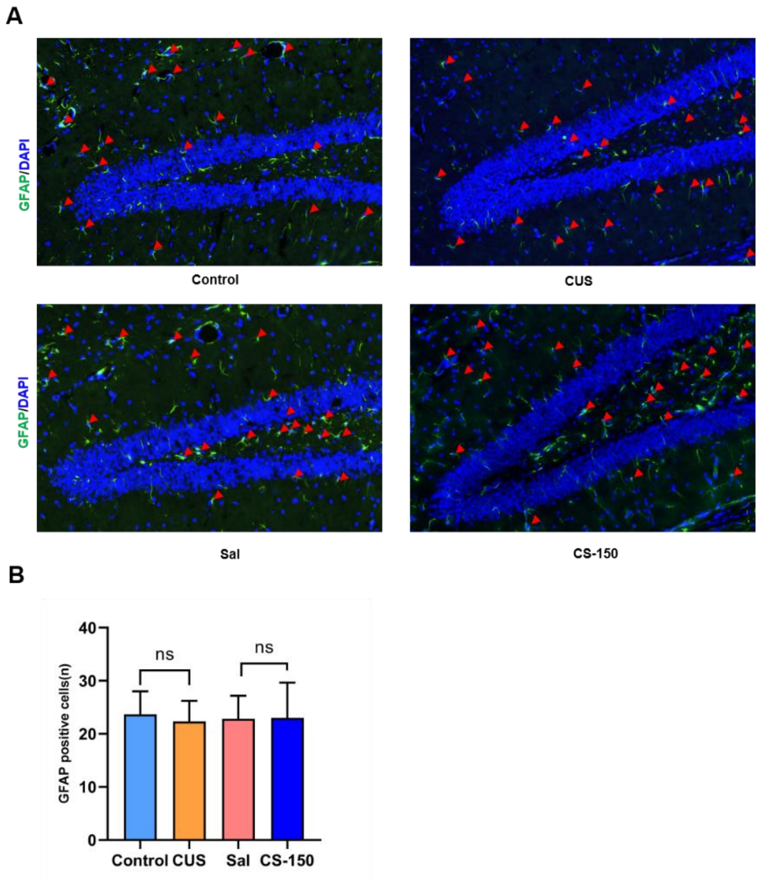

Supplementary Figure 3. CS had no effect on astrocyte in DG. (A) Images of GFAP positive cell in DG (under ×200 magnification) obtained from the immunofluorescence test. (B) The number of GFAP positive cell. The data are expressed as the mean ± standard error. ns $P > 0.05$.

Supplementary Figure 4

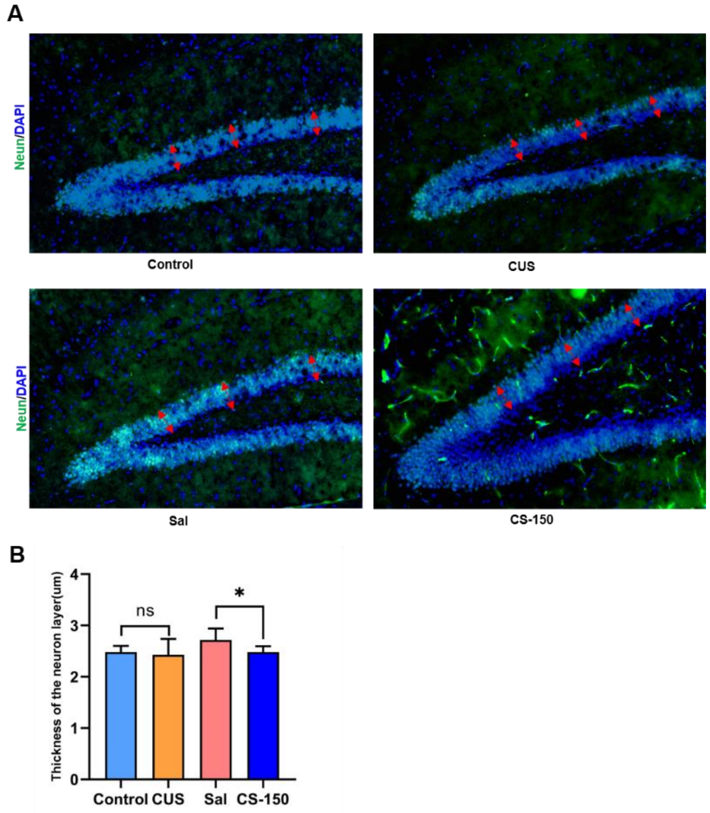

Supplementary Figure 4. CS could increase the thickness of neuron layer in the DG. (A) Images of Neun positive cell in DG (under ×200 magnification) obtained from the immunofluorescence test. (B) The thickness of neuron layer in the DG. The data are expressed as the mean ± standard error. ns $P > 0.05$. $^*P < 0.05$

Supplementary Figure 5

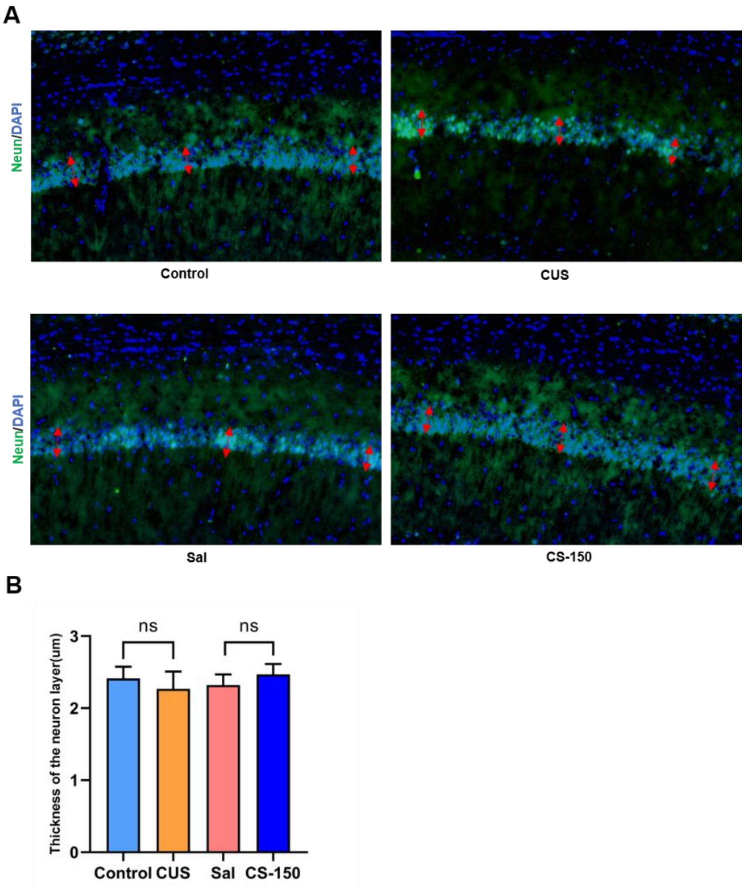

Supplementary Figure 5. CS had no effect on the thickness of neuron layer in the CA1. (A) Images of Neun positive cell in CA1 (under ×200 magnification) obtained from the immunofluorescence test. (B) The thickness of neuron layer in the CA1. The data are expressed as the mean ± standard error. ns $P > 0.05$.

Supplementary Figure 6

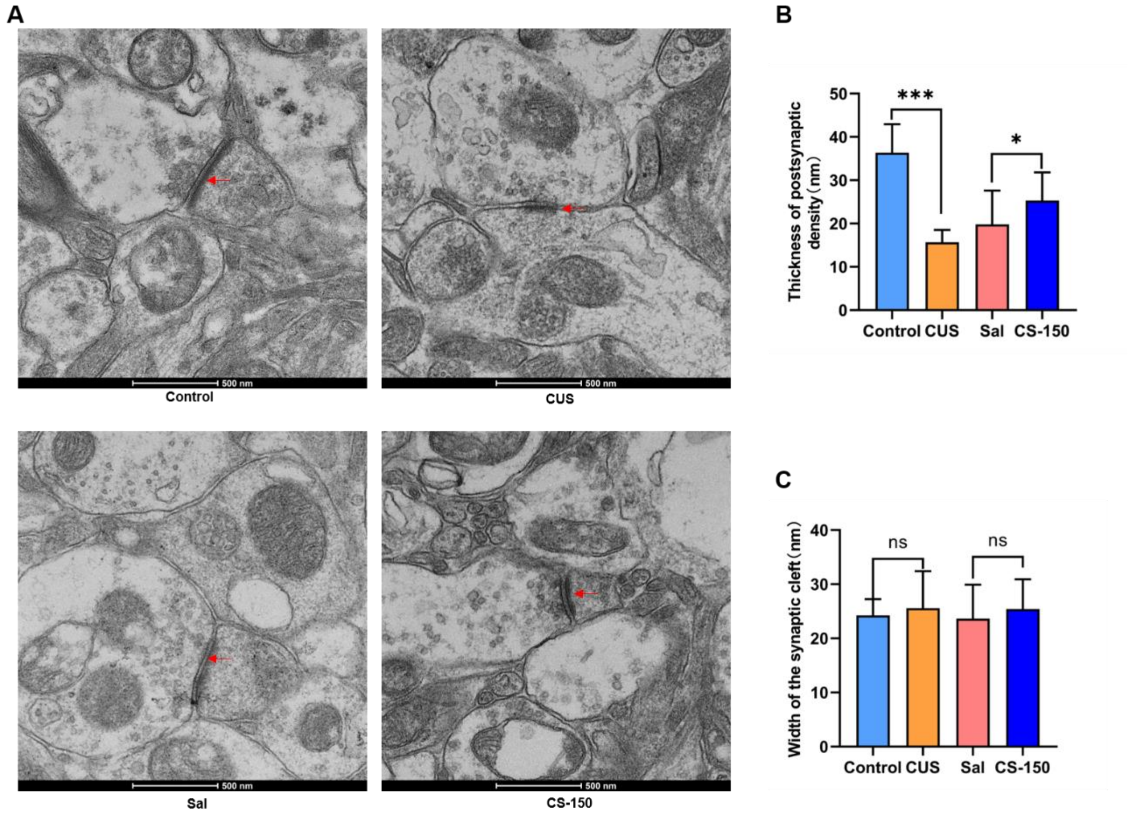

Supplementary Figure 6. CS could change the synaptic ultrastructure. (A) Synaptic ultrastructure of mPFC under ×60,000 magnification. (B) Thickness PSD in the HIP. (C) Synaptic cleft width in the HIP. The data are expressed as the mean ± standard error. *P < 0.05, ***P < 0.001, ns P > 0.05.

Supplementary Table 1 Compound content in CS extract

Compound	CS Quality(mg)	Appearance time(min)	Peak area	Content (%)
Chlorogenic acid	1.0029	10.395	235912	0.24
Hypericin	1.0029	21.503	1124549	0.27
Isoquercetin	1.0029	22.375	226032	0.056
Astragalin	1.0029	29.68	169478	0.047
Quercetin	1.0029	30.174	59052	0.017
Kaempferol	1.0029	39.071	84844	0.011
Isorhamnetin	1.0029	39.055	84640	0.011

Supplementary Table 2 Chronic unpredictable stress procedure

Time	Chronic unpredictable stress	Notes
Day 1	water deprivation for 24 h	-
Day 2	food deprivation for 24 h	-
Day 3	cold swim at 4°C for 30 min	-
Day 4	6-h cage tilt (45°)	-
Day 5	overnight illumination	-
Day 6	restraint stress for 2 h	mice were placed in 50 ml centrifuge tube with opening in one corner allowing free respiration but restricting any movement.
Day 7	stroboscopic stimulus	150 flashes/min, 200 Lumen
Day 8	a soiled cage environment	a soiled cage environment (200 mL water in 100 g sawdust bedding
Day 9	water deprivation for 24 h	-
Day 10	food deprivation for 24 h	-
Day 11	cold swim at 4°C for 30 min	-
Day 12	6-h cage tilt (45°)	-
Day 13	overnight illumination	-
Day 14	restraint stress for 2 h	mice were placed in 50 ml centrifuge tube with opening in one corner allowing free respiration but restricting any movement.
Day 15	stroboscopic stimulus	150 flashes/min, 200 Lumen
Day 16	a soiled cage environment	a soiled cage environment (200 mL water in 100 g sawdust bedding
Day 17	water deprivation for 24 h	-
Day 18	food deprivation for 24 h	-
Day 19	cold swim at 4°C for 30 min	-
Day 20	6-h cage tilt (45°)	-
Day 21	overnight illumination	-

Supplementary Table 3 the information of antibodies utilized in this study

Antibodies	Source	Identifier	Dilution
NF-κB (Rabbit)	Abcam (Cambridge, UK)	Ab194926	1:1000
COX-2 (Rabbit)	Abcam (Cambridge, UK)	Ab179800	1:1000
NLPR3 (Rabbit)	Abcam (Cambridge, UK)	Ab263899	1:1000
NRF2 (Mouse)	Proteintech Group (Wuhan, China)	16396-1-AP	1:1000
HO-1 (Rabbit)	Abcam (Cambridge, UK)	Ab189491	1:1000
β-actin (Mouse)	Beyotime (Shanghai, China)	AF0003	1:3000
GAPDH (Mouse)	Proteintech Group (Wuhan, China)	60004-1-Ig	1:10000
Neun (Mouse)	Proteintech Group (Wuhan, China)	66836-1	1:200
GFAP (Mouse)	Beyotime (Shanghai, China)	AG259-1	1:100
IBA-1(Rabbit)	Proteintech Group (Wuhan, China)	10904-1	1:200
HRP-labeled Goat Anti-Rabbit IgG(H+L)	Beyotime (Shanghai, China)	A0208	1:5000
HRP-labeled Goat Anti-Mouse IgG(H+L)	Beyotime (Shanghai, China)	A0216	1:5000
Alexa Fluor 488-labeled Goat Anti- Rabbit IgG (H+L)	Beyotime (Shanghai, China)	A0423	1:200
Alexa Fluor 488-labeled Goat Anti- Mouse IgG (H+L)	Beyotime (Shanghai, China)	A0428	1:200
